# Therapeutic update on oral potassium exchange resin use in chronic kidney disease patients: a systematic review of randomized controlled clinical trials

**DOI:** 10.3389/jpps.2023.11892

**Published:** 2023-12-20

**Authors:** Jaclyn Gruver, Akram Al-Makki, Brian Shepler

**Affiliations:** ^1^ Department of Pharmacy Practice, Purdue University College of Pharmacy, West Lafayette, IN, United States; ^2^ Nephrology Clinic, Indiana University Health Arnett, West Lafayette, IN, United States

**Keywords:** chronic kidney disease, hyperkalemia, sodium polystyrene sulfonate, patiromer, sodium zirconium cyclosilicate

## Abstract

Hyperkalemia is a common electrolyte disorder in patients with chronic kidney disease (CKD) that increases in prevalence with the decline of glomerular fltration rate (GFR). Another risk of hyperkalemia is the use of renin-angiotensin-aldosterone system inhibitors (RAASi) and/or mineralocorticoid receptor antagonists (MRAs) in managing CKD and proteinuria. The treatment of chronic hyperkalemia is challenging especially for outpatients. Treatment options for hyperkalemia include the potassium exchange resins of which two new potassium binders, Patiromer Sorbitex Calcium, and Sodium Zirconium Cyclosilicate (SZC) have demonstrated their clinical efficacy in reducing serum potassium with a positive safety profile. The old potassium exchange resin sodium polystyrene sulfonate (Kayexalate™) has some negative side effects including colonic necrosis, hypomagnesemia, and hypernatremia. In this review and literature search, we compare the available oral potassium exchange resins, highlight their advantages and disadvantages and comment on efficacy and safety parameters specifically in CKD patients.

## Introduction

Hyperkalemia is a common problem encountered by clinicians who manage patients with chronic kidney disease (CKD). While end stage kidney disease (ESKD) patients receiving hemodialysis can have potassium managed through the dialysis process, CKD stage 3 and 4 patients often must rely on other means to control hyperkalemia such as dietary regulation and medical management. Patients with CKD are often on medications that can contribute to hyperkalemia such as angiotensin converting enzyme inhibitors (ACE-I) and angiotensin receptor blockers (ARB) for hypertension and proteinuria [1].

Many therapies can be employed for the medical management of hyperkalemia including insulin, albuterol, sodium bicarbonate, diuretics, and potassium exchange resins. Outpatient therapy for CKD patients further limits the options to diuretics, which may be minimally effective depending on the degree of kidney function remaining and the patient’s ability to produce urine, sodium bicarbonate, which is typically not effective in CKD patients, and oral potassium exchange resins [2].

There are three commercially available potassium exchange resins in the United States (Table 1). Until recently, there was only one used in practice, sodium polystyrene sulfonate (Kayexalate™). Originally approved by the Food and Drug Administration (FDA) in 1958, this early cation exchange resin only needed to show proof that it was safe, not necessarily effective at the time [7]. The primary mechanism of action for sodium polystyrene sulfonate (Kayexalate™) was exchanging sodium for potassium in the gastrointestinal tract where potassium became bound and was excreted from the body via the feces. Sodium polystyrene sulfonate (Kayexalate™) came with some negative side effects including colonic necrosis, hypomagnesemia, and hypernatremia [8].

**TABLE 1:** Comparison of available potassium exchange resins [3–6].

	Sodium polystyrene sulfonate (Kayexalate™)	Patiromer (Veltassa™)	Sodium zirconium cyclosilicate (Lokelma™)
FDA approved indication	Treatment of hyperkalemia	Treatment of hyperkalemia	Treatment of hyperkalemia
Mechanism of action	Cation exchange resin, potassium ions are exchanged for sodium ions	Cation exchange polymer, potassium ions are exchanged for calcium	Potassium ions are exchanged for hydrogen and sodium ions
Sodium content	1 g contains approximately 100 mg of sodium	N/A	5 g dose contains approximately 400 mg of sodium
Site of action	Large intestine	Lumen of gastrointestinal tract, longest acting in colon	Lumen of gastrointestinal tract
Onset of action	2–24 h	7 h	1 h
Starting Dose	15 g QD to QID	8.4 g once daily	10 g TID for up to 48 h
Route of administration	Oral; Enema	Oral	Oral
Drug interactions	Separate from other orally administered drugs at least 3 h before or after	Separate from other orally administered drugs at least 3 h before or after	Separate from other orally administered drugs at least 2 h before or after
Adverse effects	Gastric irritation, anorexia, nausea, vomiting, constipation, hypokalemia, hypocalcemia, sodium retention, colonic necrosis	Constipation, hypomagnesemia, diarrhea, nausea, abdominal discomfort, flatulence	Edema, hypokalemia

FDA, Food and Drug Administration; QD, daily; TID, three times per day; QID, four times daily.

Recently two new potassium exchange resins have been approved and are currently used in practice. Patiromer (Veltassa™) was approved in 2015 and uses a calcium-based exchange resin instead of a sodium exchange [9]. The most recent addition to the class of oral potassium exchange resins is sodium zirconium cyclosilicate (Lokelma™) [10]. While there are other published reports on the use of these agents for treating hyperkalemia, the focus of this review is to compare the available oral potassium exchange resins, highlight their advantages and disadvantages and comment on efficacy and safety parameters specifically in CKD patients.

## Methods

**FIGURE 1:**
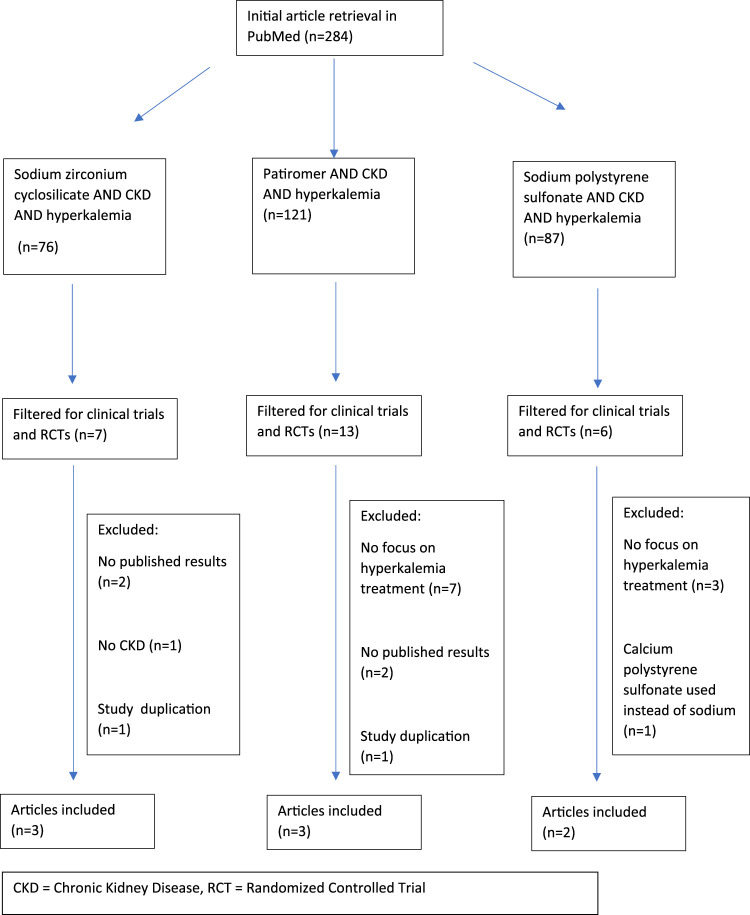
Prisma flow chart for study collection and exclusion.

Search methods followed the Preferred Reporting Items for Systematic Reviews and Meta-Analyses (PRISMA) guidelines. A literature search was performed with PubMed using the search terms “chronic kidney disease” and “hyperkalemia” with each of the following drugs: sodium zirconium cyclosilicate, patiromer, and sodium polystyrene sulfonate. Results were filtered for clinical trials and randomized controlled trials in humans and published in the English language. No specific date range was specified. Duplicate clinical trials were excluded as were any trials that did not 1) include patients with CKD, 2) provide published results, and 3) specifically address the impact on potassium serum concentrations as a primary endpoint. Figure 1 depicts the exclusion criteria and process utilized for the selection of papers in this review.

## Results

Using the methods described above, 284 studies were identified and eight were included in this review. All three commercially available products are effective at lowering serum potassium concentrations in CKD patients. Table 2 provides a summary of the clinical trials reviewed and their associated end points [11–18].

**TABLE 2:** Selected clinical trial summaries.

Authors, year	Study designs and objectives	Patients	eGFR (mL/min/1.73 m^2^)	Treatment drug and dose	Outcomes
Sodium polystyrene sulfonate (Kayexelate™)					
[11]	RCT	97	15->90	SPS: 5 g TID x 3 days	The mean potassium level at baseline was 5.8 ± 0.26 in the calcium polystyrene sulfonate group, and it lowered to 4.8 ± 0.5. The mean potassium level at baseline for the sodium polystyrene sulfonate group was 5.8 ± 0.6 and lowered to 4.3 ± 0.53
	Obj: compare the safety and efficacy of CPS and SPS in patients with CKD and hyperkalemia	CPS: 50		CPS: 5 g TID x 3 days	Participants did not develop a change in other electrolytes, weight gain, or edema
		SPS: 47			Adverse effects included abdominal pain, nausea, and anorexia
					The study showed both CPS and SPS are effective in treating hyperkalemia
[12]	RCT-Obj: evaluate the efficacy of SPS in outpatients with CKD and mild hyperkalemia	33	<40	SPS: 30 g QD x 7 days	Patients in the SPS group had an average decrease in potassium of 1.25 ± 0.56 mEq/L, compared to patients in the placebo group who had an average decrease in potassium of 0.21 ± 0.29 mEq/L (CI −1.37 to −0.71; *p* < 0.001). Patients achieving normokalemia at the end of the study period (3.5 to <5 mEq/L) between the two groups was not statistically significant (*p* = 0.07)
		SPS: 16			SPS is effective for treatment of mild hyperkalemia in outpatients with CKD.
		Placebo: 17			
Patiromer (Veltassa™)					
[13]	Phase 2, RCT	306	15–60	Patiromer	Reduction in potassium was seen in approximately 48 h for both mild and moderate hyperkalemia groups (*p* < 0.001). At 4 weeks, in those with mild hyperkalemia, the reduction in potassium from baseline was 0.35 mEq/L (CI 0.22–0.48) for those receiving 8.4 g per day, 0.51 mEq/L (CI 0.38–0.64) for those receiving 16.8 g per day, and 0.55 mEq/L (CI 0.42–0.68) for those receiving 25.2 g per day. At 4 weeks, in those with moderate hyperkalemia, the reduction in potassium from baseline was 0.87 mEq/L (CI 0.60–1.14) for those receiving 16.8 g per day, 0.97 mEq/L (CI 0.7–1.23) for those receiving 25.2 g per day, and 0.92 mEq/L (CI 0.67–1.17) for those receiving 33.6 g per day
	Obj: evaluate long term safety and efficacy of patiromer in CKD patients with HTN, diabetes, and hyperkalemia	Mild hyperkalemia: 222		Mild hyperkalemia: 4.2 g BID, 8.4 g BID, 12.6 g BID	Through week 52, the proportion of patients with potassium levels within the target range (3.8–5.0 mEq/L) ranged from 83.1 percent to 92.7 percent in patients with mild hyperkalemia and from 77.4 percent to 95.1 percent in patients with moderate hyperkalemia
		Moderate hyperkalemia: 84		Moderate hyperkalemia: 8.4 g BID, 12.6 g BID, 16.8 g BID	Adverse effects reported include worsening of CKD, hypomagnesemia, worsening of hypertension, constipation, and diarrhea
[14]	2 phase study with 4 weeks treatment phase followed by randomized withdrawal (OPAL-HK)	237	15–60	Patiromer	76% of patients reached potassium serum concentrations of 3.8 to <5.1 mmol per liter. A recurrence of hyperkalemia occurred in 60% of the placebo group compared to only 15% in the patiromer group (*p* < 0.001)
	Obj: evaluate treatment efficacy and recurrence of hyperkalemia			4.2 g BID or 8.4 g BID	
[15]	RCT	63	<60	Patiromer	The average change in potassium at the end of the study from baseline was −0.13 mEq/L. At the end of the study, 57 (90.5 percent) participants had a potassium level between 3.5 and 5.5 mEq/L (CI 0.84–0.964). All 63 patients were able to be titrated to the maximum dose of spironolactone
	Obj: evaluate the effectiveness of individualized titration of patiromer, safety and tolerability of patiromer in CKD patients with HF			8.4 g BID	Adverse events included worsening of renal function, abdominal discomfort, flatulence, headache, and hypertension
				Allowed to be titrated up to 50.4 g/day and down to 0 g/day	
Sodium zirconium cyclosilicate (Lokelma™)					
[16]	Phase 2, RDBPCT	90	30–60	Sodium zirconium cyclosilicate	Serum potassium was reduced in the 3 g and 10 g group with statistical significance (*p* 0.048 and *p* < 0.0001 respectively)
	Obj: Safety and efficacy in CKD			0.3 g TID (*n* = 12)	
				3.0 g TID (*n* = 24)	
				10 g TID (*n* = 24)	
[17]	Phase 3b RDBPCT	196	CKD 5 HD	Sodium zirconium cyclosilicate	A significantly higher number of patients in the treatment group did not require rescue therapy and were able to maintain their predialysis serum potassium concentrations on sodium zirconium cyclosilicate [41.2% v. 1.0% in placebo, *p* < 0.001]
	DIALIZE study			5–15 g/day	
[18]	Phase 3, open label, *post hoc* for CKD	751 eGFR <30: 289 eGFR ≥30: 453	<30	Sodium zirconium cyclosilicate	During the correction phase, 82 percent of both eGFR groups achieved normokalaemia after 24 h. By 72 h, 100 percent of those in the eGFR <30 and 95 percent of those in the eGFR ≥30 achieved normokalaemia. During the maintenance phase, at day 365, 82 percent of those in the eGFR <30 and 90 percent of those in the eGFR ≥30 achieved normokalaemia. Adverse effects were more common in the eGFR <30 group. Common treatment adverse effects experienced by both groups included constipation, nausea, and peripheral edema
	Obj: compare safety and efficacy of SZC for hyperkalemia up to 12 months in CKD patients with a GFR <30 versus ≥30		≥30	Correction phase: 10 g TID for 24–72 h until normokalaemia	The study showed SZC normalizes potassium levels within 72 h and regardless of CKD stage normokalaemia was maintained for up to 365 days
				Maintenance phase: 5 g QD for a maximum of 12 months	

eGFR, estimated glomerular filtration rate; RCT, randomized controlled trial; RDBPCT, randomized double blind placebo controlled trial; Obj, objective; CPS, calcium polystyrene sulfonate; SPS, sodium polystyrene sulfonate; CI, confidence interval; SZC, sodium zirconium cyclosilicate; HTN, hypertension.

Two studies were selected that examined sodium polystyrene sulfonate (SPS). The first study reviewed was Nasir et al. and enrolled 97 patients with eGFR between 15 and 90 mL/min. The study compared SPS to calcium polystyrene sulfonate (CPS) (A Canadian potassium exchange resin), both dosed at 5 g three times per day for 3 days. The mean potassium concentration in the SPS group was lowered from 5.8 ± 0.6 to 4.3 ± 0.53 (no *p*-value reported for this result compared to CPS). The study reported a similar lowering effect on serum potassium concentrations between the two drugs and that side effects for were no different for the two except for incidence of nausea which was statistically higher in the SPS group (9 CPS versus 20 SPS, *p* = 0.005). The second SPS study evaluated was Lepage et al. who enrolled 33 patients with CKD (eGFR <40 mL/min) and tested SPS to placebo specifically for efficacy. Patients who received 30 g per day for 7 days of SPS experienced an average decrease in serum potassium of 1.25 ± 0.56 mEq/L compared to placebo 0.21 ± 0.29 mEq/L (*p* < 0.001).

Three studies met inclusion criteria for patiromer. The first patiromer study evaluated was by Bakris et al. and was a phase 2 randomized controlled trial of 306 CKD patients (eGFR 15–60 mL/min) who received patiromer in doses ranging from 4.2 g twice per day to 16.8 g twice per day. Patients were divided into mild hyperkalemia and moderate hyperkalemia groups. See Table 2 for specific reductions and confidence intervals. The overall potassium lowering effect in both groups was observed within 48 h and reached statistical significance (*p* < 0.001). The next patiromer study was conducted by Weir et al. and included 237 CKD patients (eGFR 15–60 mL/min). Seventy six percent of patients reached potassium concentrations between 3.8 and 5.1 mmol/L. Hyperkalemia recurred in 60% of placebo patients compared to only 15% in patiromer treated patients (*p* < 0.001). The third patiromer randomized controlled study selected was conducted by Pitt et al. and enrolled 63 CKD patients (eGFR <60 mL/min) given 8.4 g of patiromer twice daily with a titration allowance up to 50.4 g per day. The study was designed to test whether patiromer could keep the serum potassium with normal range despite the addition of spironolactone, a drug known to increase serum potassium levels but usually beneficial for hypertension in CKD patients. At the conclusion of the study, 90.5% of subjects had had potassium serum concentrations between 3.5–5.5 mEq/L (CI 0.84–0.964).

There were three sodium zirconium cyclosilicate (Lokelma™) studies identified. The first was by Ash et al. and was a randomized double-blind placebo-controlled trial following 90 CKD patients (eGFR 30–60 mL/min) who were given different doses of sodium zirconium cyclosilicate (Lokelma™) ranging from 0.3 g three times per day to 3 g three times per day to 10 g three times per day. Mean serum potassium was reduced by 0.92 ± 0.52 mEq/L in the 3 and 10 g per day groups (*p* 0.048 and *p* < 0.0001 respectively) and no adverse events were reported. In the second trial, Fishbane et al. conducted a study in 196 dialysis patients dosing sodium zirconium cyclosilicate (Lokelma™) from 5 to 15 g per day and found that more patients were able to maintain their predialysis serum potassium concentrations compared to placebo (41.2% versus 1.0% in placebo, *p* < 0.001). The last study was by Roger et al. and included 751 patients with either eGFR <30 or >30 mL/min observed over a period of 12 months. Sodium zirconium cyclosilicate (Lokelma™) was dosed at 5 g per day and by 72 h, 100 percent of the eGFR <30 mL/min group and 95 percent of the >30 mL/min group reached normokalemia.

## Discussion

This systematic review resulted in eight randomized controlled trials assessing the use of the three potassium exchange resins. Two of the trials examined sodium polystyrene sulfonate (Kayexalate™), three trials reviewed patiromer (Veltassa™) and three trials examined sodium zirconium cyclosilicate (Lokelma™). The focus of this review was on efficacy and safety of the three potassium exchange resins, both of which were important inclusion criteria for study selection. Additional studies reviewing the ability to continue the use of renin angiotensin aldosterone system inhibitors (RAASi) by using potassium exchange resins were excluded from this analysis but are mentioned below in the discussion.

Limitations for the reviewed studies included small sample sizes ranging from 33 to 306 subjects in six of the eight studies reviewed, discrepancies in dosing protocols compared to FDA package insert directions, and patient baseline demographics as described below.

### Sodium polystyrene sulfonate (Kayexalate™)

The oldest product is sodium polystyrene sulfonate (Kayexalate™). In the two studies included here, the drug is shown to be effective in lowering serum potassium in CKD patients. One study enrolled patients with estimated glomerular filtration rates (eGFR) 15–90 mL/min/1.73 m [2] and the other was more selective and only included patients with eGFRs <40 mL/min/m^2^. *p* values for significance were only provided in the Lepage et al. work [12]. The authors also noted that significantly more patients in the placebo group were receiving insulin therapy which could have affected serum potassium concentrations; however, a sensitivity analysis using multivariate regression models indicated this would not affect the outcome. Two major questions surrounding the use of sodium polystyrene sulfonate (Kayexalate™) use is the effect the sodium formulation may have on edema and blood pressure and the incidence of colonic necrosis, a potentially lethal adverse effect. Dangers of additional sodium administered to chronic kidney disease patients from sodium polystyrene sulfonate (Kayexalate™) date back to the 1960s in a report published in the Lancet indicating edema, heart failure, and increased blood pressure were concerns [19]. The 2014 study by Nasir et al did not show significant weight gain or edema with sodium polystyrene sulfonate (Kayexalate™) compared with calcium polystyrene sulphonate (Resonium Calcium), a non-sodium containing Canadian potassium exchange resin not available in the United States [11]. There was a significant increase in diastolic blood pressure however from 76.67 ± 9.3 to 82.2 ± 11.7 (*p* 0.004). Lepage et al. did not measure blood pressure as an outcome and there were no statistically significant effects on sodium concentrations. It is also important to note that no cases of colonic necrosis were observed; however, the authors indicate that the small sample size of 33 patients may have impacted their ability to accurately assess this risk [12]. The serious adverse effect of colonic necrosis with sodium polystyrene sulfonate (Kayexalate™) is rare and has been linked to use with sorbitol preparations although reports on this have been conflicting [20–22].

### Patiromer (Valtessa™)

Patiromer (Valtessa™) is the only potassium exchange resin that does not include a sodium component and is a sorbitol-calcium preparation. The trials selected for this review examined the effect on serum hyperkalemia as an end point without regard to testing the effects of other potassium altering medications. There are other clinical trials of patiromer (Veltassa™) that study the extent to which potassium sparing diuretics like spironolactone and renin-angiotensin aldosterone system inhibitors (RAASi) therapy may be continued when administered with patiromer (Veltassa™) as well as its possible effect on aldosterone [23, 24]. In the Pitt et al. study, all patients were receiving spironolactone but it was not tested as a separate variable with a placebo group and therefore remained in the inclusion group. Also of note, the dose of patiromer (Veltassa™) could be titrated up to a maximum dose of 50.4 g of patiromer (Veltassa™) which is double the maximum dose outlined in the package insert of 25.2 mg per day [15]. Another limitation of the Pitt et al. study was the inclusion of only white patients. All three studies reported a significant decrease in serum potassium concentrations. The Weir et al. study also examined recurrence of hyperkalemia after an 8 week period and demonstrated that only 15% of patiromer (Veltassa™) treated patients experienced this compared to 60% in the placebo group [14]. The most common adverse events reported across the studies was gastrointestinal upset as constipation or diarrhea. Blood pressure changes were not considered to be related to patiromer (Veltassa™) except in one case in the Bakris et al. study [13].

### Sodium zirconium cyclosilicate (Lokelma™)

Three trials met the inclusion criteria set forth for sodium zirconium cyclosilicate (Lokelma™). One was a *post hoc* analysis looking specifically at CKD patients with eGFR <30 mL/min [18]. Given that sodium zirconium cyclosilicate (Lokelma™) contains sodium like sodium polystyrene sulfonate (Kayexalate™), there is a question as to whether it would affect edema and blood pressure. Fifteen percent of patients in the <30 mL/min/1.73 m^2^ experienced hypertension and 13% experienced edema. This is an import trial to consider even though it is a *post hoc* analysis because it examines the long-term effects of sodium zirconium cyclosilicate (Lokelma™) as patients were monitored for 12 months of therapy. The other 2 trials were short by comparison at 11 weeks and 48 h. Neither were able to show any statistically significant effect on blood pressure or edema. The Roger et al. trial used a lower initial dosage but extended the period of treatment beyond what is recommended for sodium zirconium cyclosilicate (Lokelma™), and was the only trial to include participants in all stages of CKD [18].

Currently, there are no studies that directly compare all three products products head-to-head. Sullivan et al published a report in September of 2023 that examined SPS compared to Sodium zirconium cyclosilicate (Lokelma™); however, patiromer was not included and all patients with end stage kidney disease or those receiving kidney replacement therapy were excluded [25]. There is a trial KBindER (NCT04585542) that is going to evaluate the use of potassium exchange resins for acute hyperkalemia in non-CKD patients [26]. Another ongoing trial is MorphCKD which will determine if patiromer will allow for intensified RAAS blockade to reduce albuminuria [27].

## Conclusion

All three potassium exchange resins have randomized controlled trials that demonstrate their efficacy in lowering serum potassium concentrations in chronic kidney disease patients. Safety issues related to sodium containing agents include blood pressure elevation and increased edema although these were not equally observed across all studies and may warrant further investigation with larger sample sizes to confirm. Serious gastrointestinal issues may be associated with the use of the older sodium polystyrene sulfonate (Kayexalate™). Patiromer (Veltassa™) is the only non-sodium containing potassium exchange resin and has no effect on blood pressure or edema. A large head-to-head trial comparing potassium exchange resins is needed and would shed light on the efficacy and safety of these medications.
